# Cognitive Penetration and Predictive Coding: A Commentary on Lupyan

**DOI:** 10.1007/s13164-015-0254-3

**Published:** 2015-05-09

**Authors:** Fiona Macpherson

**Affiliations:** Centre for the Study of Perceptual Experience, Department of Philosophy, University of Glasgow, Glasgow, UK

The main aim of Lupyan’s paper (this volume) is to claim that perception is cognitively penetrated and that this is consistent with the idea of perception as predictive coding. In these remarks I will focus on what Lupyan says about whether perception is cognitively penetrated, and set aside his remarks about epistemology.

I have argued (2012) that perception can be cognitively penetrated and so I am sympathetic to Lupyan’s overall aim of showing that perception is cognitively penetrable. However, I will be critical of some of Lupyan’s reasoning in arguing for this position. I will also call for clarification of that reasoning. First, I will discuss what is meant by cognitive penetration and, in light of this, the sort of evidence that can be used to establish its existence. Second, I will question whether Lupyan establishes that all cases of cross-modal effects are cases of cognitive penetration. In doing so, I will suggest a form of cognitive penetration that has heretofore not been posited, and give an example of how one might test for its existence. Third, I will question whether Lupyan puts forward convincing evidence that categorical knowledge and language affect perception in a way that conclusively shows that cognitive penetration occurs. Fourth, I will closely examine Lupyan’s reply to the argument against cognitive penetration from illusion. I show that his reply is not adequate and that he misses a more straightforward reply that one could give. Fifth, I briefly concur with the remarks that Lupyan makes about the role of attention with respect to cognitive penetration. And finally, sixth, I spell out exactly what I think the relationship is between the thesis that predictive coding is the correct model of perception and the thesis that there is cognitive penetration.

## Defining Cognitive Penetration

What is cognitive penetration? Lupyan says that he accepts Pylyshyn’s (1999) definition of cognitive penetration: a perceptual system is cognitively penetrable if “the function it computes is sensitive, in a semantically coherent way, to the organism’s goals and beliefs, that is, it can be altered in a way that bears some logical relation to what the person knows” (1999: 343). Unlike Lupyan, Pylyshyn holds that there is no penetration of early vision: “A major portion of vision, called the early vision system, does its job without the intervention of knowledge, beliefs or expectations, even when using that knowledge would prevent it from making errors.” (1999: 414). The early visual system is defined functionally, as a system that takes attentionally modulated signals from the eyes (and perhaps some information from other sensory modalities) as inputs, and produces shape, size and colour representations—representations of visual properties—as output.

Somewhat in contrast to Pylyshyn, when philosophers have been interested in cognitive penetration they have often been more interested in whether perceptual experience—a conscious perceptual state of people—can be penetrated, rather than whether early vision can be penetrated. That is, they are interested in whether perceptual experience is sensitive, in a semantically coherent way, to the person’s beliefs, desires, goals or other cognitive states.

The first of two issues that I wish to bring up concerning how one should define cognitive penetration is whether such definitions spell out sufficient conditions for cognitive penetration. In other words, is it sufficient for cognitive penetration that beliefs, desires, or other cognitive states affect early vision or perceptual experience in a “semantically coherent” way?

One might think that it is not. Consider the following example: You believe that aliens might invade the planet. This belief causes you immense stress and a migraine ensues. Your migraine causes you to experience flashing lights in the periphery of your vision. The experience as of flashing lights semantically coheres with your belief that aliens might land: for they plausibly would do in spaceships with flashing lights. So, according to the definition we are considering, this would be a case of cognitive penetration.

But, arguably, this is not a case of cognitive penetration, just a case of a migraine. Why might one think that? The reason is that one might think that although the migraine causes a perceptual experience of flashing lights it is purely by chance that this experience is semantically related to the belief that caused the migraine. After all, were it an exam that caused one stress and the subsequent migraine, one would still have had the perceptual experience of flashing lights—not a perceptual experience semantically related to the belief about one’s exam.

In order to come up with a better definition of cognitive penetration, then, one might insist on further conditions that have to be fulfilled to rule out this kind of case. There are various options that one could consider. One is to add the condition that, in addition to a semantic relation between the content of the cognitive state and the perception, any intermediate state that does the penetrating—in the example above, the migraine state—has to have the content that it does because the cognitive state that causes it has the content that it has. Another is to add the condition that there has to be a direct transfer of content from the cognitive state via any intermediate states into the perceptual state. There are also others that one could consider.

Lupyan doesn’t address this issue. One might be tempted to say that this is a general issue that needs to be addressed by everyone working on cognitive penetration that and more work needs to be done on this topic in general. However, as we will see later in this paper, this may be an issue that is more pressing for Lupyan’s approach to defending cognitive penetration than other approaches. I will return to this topic in section 2.

A second definitional issue that arises concerns Lupyan’s acceptance of Pylyshyn’s definition of cognitive penetration. Recall that his definition was that cognitive penetration took place when early vision was affected by cognition, rather than perceptual experience. Lupyan does not discuss the relationship between early vision and visual experience, but he needs to do so (as does Pylyshyn). The reason he needs to do so is because a lot of the evidence that Lupyan cites in favour of (and Pylyshyn’s cites against) the effects of cognition on early vision involves considering evidence about whether perceptual experience is altered by a subject’s cognitive states. Evidence about whether perceptual experience is altered can only be evidence about whether the processing in the early visual system is altered if one thinks that changes in the former indicate changes in the latter. But such a thesis must be argued for. And without such an argument, it is hard to see how someone arguing for cognitive penetration can take the nature of perceptual experience as evidence for it, as Lupyan (and indeed Pylyshyn) often do.

Here is one example in which Lupyan takes evidence about perceptual experience to show something about the cognitive penetration of early vision. Lupyan claims that uttering the word “zebra” makes all and only pictures of zebra visible—pictures that would otherwise not be visible due to continuous flash suppression. Evidence for this is gathered from subjects’ reports about what they perceptually experience. How does that show that cognitive penetration of early vision occurs? It does so only if one assumes that the having of a visual experience is evidence for changes to what is going on in early vision, rather than changes to what goes on after early vision. Examples of these kinds abound in Lupyan’s paper, such as the illusions cases and the cross-modal cases.

A particular problem for Lupyan (but not Pylyshyn) with respect to this issue is that Lupyan buys into the predictive coding model of perception. As stated previously, evidence about whether perceptual experience is affected by cognition can only be evidence about whether the processing in the early visual system is affected by cognition if one thinks that changes in the former indicate changes in the latter. If one thought, for example, that the output of early vision was identical to perceptual experience, or that it determined the nature of perceptual experience, or that it determined the nature of some aspects of perceptual experience (as arguable Pylyshyn does), then one could use this to argue for the dependence of perceptual experience on the processing that takes place in early vision. However, on the predictive coding model of perception, the idea that perceptual experience or any elements of perceptual experience are generated by early visual processing—at least generated by that alone—is one that is squarely rejected. Let me explain why.

On the predictive coding model of perception, the brain produces top-down generative models or representations of the world that are predictions of how the world is. These draw on many high-level processing levels including cognitive states such as beliefs, desires, and goals. Those representations are then modified bottom-up by incoming, lower-level processing, top-down by knowledge, belief, expectation, and prior experience, and perhaps sideways by other sensory modalities. The aim in so doing is to reduce global prediction error. Any and all bottom-up and top-down processes will be used whenever they would reduce prediction error. Different versions of the theory have different detailed accounts of how this prediction error minimisation occurs. Lupyan doesn’t argue for this view, but assumes it. In doing so, he tries to show that on such a model we should expect cognitive penetration.

Andy Clark explains what determines visual experience (often called a “visual percept” in the psychological literature) on the predictive coding theory:

a visual percept is determined by a process of prediction operating across many levels of a (bidirectional) processing hierarchy, each concerned with different types and scales of perceptual detail. All the communicating areas are locked into a mutually coherent predictive coding regime, and their interactive equilibrium ultimately selects a best overall (multiscale) hypothesis concerning the state of the visually presented world. This is the hypothesis that “makes the best predictions and that, taking priors into consideration, is consequently assigned the highest posterior probability” (Hohwy et al. 2008, p. 690). Other overall hypotheses, at that moment, are simply crowded out: they are effectively inhibited, having lost the competition to best account for the driving signal. (Clark 2013: 185)

In short, a perceptual experience is identified with the winning—surviving—best prediction or representation of how the world is. And that is generated by high-level and low-level processing, including cognitive states and processes.

If this is the model of visual experience that Lupyan would subscribe to whilst holding the predictive coding model of perception (I say this in the conditional form for he is not explicit about it) then he cannot hold that it is early visual processing that determines the nature of visual experience. Given this, I do not see how he can hope to determine that cognitive penetration, understood as penetration of early visual processing, can be established by demonstrating that perceptual experience is modified by cognitive states. In other words, if Lupyan is interested in establishing the falsehood of Pylyshyn’s thesis that early vision is not cognitively penetrated, then he has not explained why he thinks that evidence about the nature of perceptual experience could help one determine this, given that on the predictive coding model of perception, perceptual experience is determined by both early visual processing together with higher-level (including cognitive) states.

I suspect that Lupyan is actually concerned about whether perceptual experience is cognitively penetrated by cognition, and so he should not follow Pylyshyn and use his definition of cognitive penetration.

## The Evidence About Cognitive Penetration from Cross-Modal Interaction

Recall that Lupyan accepts the predictive coding model of perception and wishes to show that it is compatible with cognitive penetration. Lupyan claims that cross-modal processing counts as cognitive penetration on the predictive coding model. Cross-modal processing occurs when two sensory systems interact. For example, information processed in one modality, say hearing, might affect information processed in another modality, say vision, or an experience had in one modality, say vision might affect the experience had in another modality, say, touch. Examples of cross-modal processing that Lupyan cites include the McGurk effect, in which the lip movements that one sees affects the sound that one seems to hear, and the sound flash illusion, in which the number of beeps that one hears affects the number of visual flashes that one seems to see.

On a view of perception at odds with the predictive coding model, there is no good reason to think of cross-modal processing as a form of cognitive penetration. Cross-modal processing would be an instance not of cognition affecting perception but of different forms of perception affecting each other. However, Luypan claims that this is not the case if one accepts the predictive coding model: cross-modal processing counts as cognitive penetration. The reason that he gives for thinking this is that perception is: “not a fixed, reflexive system where by some inexplicable quirk [of] vision can be modulated by sound or touch, but rather a highly flexible system where these modulations can be explained as ways of lowering the overall prediction error.”

Is Lupyan right to think that, if we accept the predictive coding model, cross-modal processing counts as cognitive penetration? One might think that some instances of cross-modal processing do, for cross-modal effects are compatible with the existence of cognitive penetration. Lupyan does not provide examples of such cases. Nonetheless, reflection on the matter reveals ways in which cases of cross-modal processing could also count as being cases of cognitive penetration. Here are two examples:

1. If processing in one modality is affected by cognition, and the subsequent product then affects another modality in a suitable way, then the cross-modal influence could be an instance of cognitive penetration. It would be a case of indirect cognitive penetration via an intermediate perceptual state.^1^ This form of cross-modal effect counts as an instance of cognitive penetration simply because a prior instance of cognitive penetration has occurred: penetration of the sensory modality that goes on to have the effect on a second sensory modality.
2. If a cross-modal effect occurs only because the subject’s cognitive system is in a particular state, then the cross-modal influence could be an instance of cognitive penetration. (Note that this case does not rely on a prior act of cognitive penetration, as (1) above does.)

(2) is a form of cognitive penetration that I don’t believe has been considered before. Let me therefore explain in more detail the sort of case that I have in mind. Take as an example a classic cross-modal effect: the sound induced flash illusion that Lupyan discusses in his paper. Shams et al. (2000) discovered that when a single visual flash is accompanied by two auditory beeps, people frequently report that they experience two visual flashes. Suppose, for the sake of argument, that if the two beeps seem to originate from the same location as the visual flash one visually experiences a double flash, but one experiences only one visual flash if the two beeps seems to emanate from a location some distance from the location of the visual flash—if one is not provided with any further information. Let me make it clear that I don’t know whether this location effect occurs. I am simply asking you to suppose that it does. Now suppose that we told the subjects that the beeps which emanate from a location different to that of the visual flash are connected in some way to the flash. For example, suppose one told the subjects that the beeps that emanated some distance from the location of the visual flash were in fact produced by speakers connected to the computer that generated the visual flash and that the computer always produces beeps when it produces flashes. Now suppose that we exposed those subjects who were told of this connection again to the visual flash and the beeps located some distance from it, and suppose further that they now had an experience as of a double flash. And suppose that more generally we determined that subjects experienced the double flash when the sound source was at a distance from the visual flash when and only when they believed that there was a connection between them. This would be evidence that the cross-modal illusion occurred when the visual flash and the beeps were at different locations only when the subject was in a particular cognitive state: that of believing that the visual flashes and the beeps were connected in the way just specified. It would be evidence that cognitive penetration of a sort had occurred: the modification of experience by cross-modal effects only because of the presence of the right cognitive state. This is an interesting form of cognitive penetration that to my knowledge has not been considered before.

(2) is a very specific form of cognitive penetration compatible with cross-modal effects. One would hesitate from thinking that it occurred every time there was a cross-modal effect. I think that the best strategy that Lupyan has for arguing that cross-modal effects are always instances of cognitive penetration, as he seems to want to argue, is to show that cognition always affects perception. So he might try to argue that the following is always the case if the predictive coding model of perception is true:

(3) When processing in one modality affects processing in another modality, prior cognitive states also affect that perceptual processing.

(3) is more general and less specific than (1), and would include cases of (1) as instances. But should we accept (3), and should we accept it even if we believe the predictive coding model of perception? There is reason to resist it. It seems plausible that if predictive coding were true, sometimes predictions would be generated by high-level processes, but nonetheless, processes that are below the level of cognition (subpersonal processes) might, on some occasions, generate the predictions too. If so, on some occasions, cognition would not feed into perception. In my opinion, it would be hard to argue that cognition must always contain priors about how the world is, and that predictions are always to some extent generated by cognition. What could guarantee that cognition must always have relevant hypotheses or information about the nature of the world?

In response to this, one might try to argue that, even when cognition has no priors about the way the world is, it still does, in a sense, influence perception, so that, in a minimal way, cognition always has an effect on perception. This would just be the minimal effect of feeding no information into the perception system or feeding in information to the effect that no information could be usefully added by cognition on that occasion. I think that this is the only strategy that could be used to try to make the case that every instance of perception was cognitively penetrated or that every instance of a cross-modal effect was also one of cognitive penetration.

Consider this strategy further. When the cognitive system lacked any information or priors about the way the world was, would it be the case that cognitive penetration was occurring? Suppose that perception was sensitive in the appropriate way to the information that the cognitive system had—when it had it—so that the “function it computes is sensitive, in a semantically coherent way, to the organism’s goals and beliefs, that is, it can be altered in a way that bears some logical relation to what the person knows” (1999: 343). Clearly such a system would be *penetrable*. Still, we can ask, would it be *penetrated* on the occasions where there was a lack of information? Would we say that there was an “intervention of knowledge, beliefs or expectations” (1999: 414) on perception, which is required for cognitive penetration?

I am not sure how to answer this question. In such a case, in one sense, there is a lack of intervention by the cognitive system because it doesn’t feed information into the perceptual system. This makes one tempted to say that there is a lack of cognitive penetration in this case.

In another sense, one might think that the cognitive system still exhibits an influence over the perceptual system when it has no information to contribute, for its contributing no information, rather than some information, will affect what processing the perceptual system carries out, for that the system would carry out different processing if the cognitive system had some information to contribute. So, in this sense, one might be tempted to say that there is cognitive penetration in such a case. Note that, if we allow this to count as a case of cognitive penetration, then the semantic or logical connection between the content of the cognitive system and the content of the perceptual system that is required to be present in cases of cognitive penetration must be exceptionally minimal. For the content penetrating is the absence of content in such cases. If one held these cases were ones of cognitive penetration then the problem that I discussed in section one—that of the definition of cognitive penetration as involving suitable links between the content of cognition and the content of perception not being sufficient—would be even more pressing. This is because the more minimal one requires these links to be the more the definition will let in cases of the type like the migraine case that are intuitively not cases of cognitive penetration.

Whether we should think of these kinds of cases in which the cognitive system contributes no content or information to perception as being cases of cognitive penetration is unclear to me. I wonder if Lupyan can convince us to adopt the view that there is cognitive penetration in such cases.

## Penetrability of Visual Processing by Categorical Knowledge and Language

Lupyan claims that categorical knowledge and language can affect our perception and thus, he claims, that perception is cognitively penetrated. Are the alleged categorical knowledge and language effects on perception really ones from cognition? One can question whether they are.

Consider two cases that Lupyan cites as instances of cognitive penetration by categorical knowledge and language:

(i) hearing the word “brick”, for someone who has learned the meaning of the word, provides top-down activation of brick-like properties in the visual system enabling “faster detection of a reddish rectangular object hurling our way”.
(ii) Hearing the word “zebra” makes all and only stimuli of pictures of zebra visible, which otherwise would not be visible due to continuous flash suppression (Lupyan and Ward 2013).

In these cases, Lupyan claims that the cognitive system is affecting perception. He thinks that states of the cognitive system, such as the state of one’s concept of a brick or a zebra being primed, or the beliefs that one has about bricks and zebras, penetrate perception giving one a perceptual experience that one would not have had—or not have had at least at that moment—were it not for the impact of the cognitive system on perception.

However, there is another alternative explanation that Lupyan does not consider. One model of word perception is that there are distinctive lexical and semantic word processing systems in the brain. The former is taken to be perceptual and non-cognitive; the latter is taken to be cognitive. There is evidence that the lexical system is independent of the semantic system. For example, Schacter (1992) argues that there is a visual word form system that:

process[es] and represent[s] information about the form and structure, but not the meaning and other associative properties, of words… Evidence that such a system operates at a presemantic level is provided by studies that have focused on brain-damaged patients who maintain relatively intact abilities to read words yet exhibit little or no understanding of them (1992: 245)

Likewise, Fodor (1983) argues that there can be non-semantic lexical processing, and connections between a word in the lexical system and words stored in a lexical memory store can be made without semantic meaning playing a role. For example, he cites an experiment by Swinney (1979) in which visual exposure to an ambiguous word, in a context that implied one disambiguation rather than another, facilitated subjects in correctly determining whether a string of letters was a word or a non-word in cases where what was presented was a word related in meaning to either disambiguation of the ambiguous word. For example, exposure to the word “bug” in a context where it refers to a small insect, rather than a device for surveillance, facilitated not only recognition of “ant” as a word, rather than a non-word, but also “spy”.^2^

If this is right, then the brick and the zebra cases could be explained without involvement of the cognitive system. Rather, the tokening of the words in the lexical module might trigger or prime various perceptual representations without belief, knowledge or concepts—hence cognition—being involved. For it could be the case that connections between words in a lexical module and certain perceptual representations have been previously established—connections which do not depend on processing of the semantics of the lexical items. For example, the word “brick” might prime visual representations of red rectangular objects, and the word “zebra” might prime representations of black and white striped forms of a certain animal-like form. That such visual representations are primed might also explain why hearing “zebra” makes it more difficult to detect unrelated objects like pumpkins. In short, Lupyan has not shown that the effects he discusses are not inter-modular (lexical – perception) non-cognitive effects, rather than effects of cognition on perception. But he must rule out this possibility in order to establish that perception is penetrated by categorical knowledge and the semantics of language, and hence cognition.

Indeed, when Lupyan describes the zebra case, much of the language that he uses is in line with the alternative non-cognitive interpretation of the case. For example, he claims that, “The results showed that *simply hearing* a word was sufficient to unsuppress and make visible otherwise invisible images” (my emphasis). Note that he does not say that hearing *and understanding* the word is sufficient for the effect. Hearing the word alone, he says, is sufficient. Moreover he describes the effect as occurring in virtue of the “label” “zebra”.

One way that Lupyan could reply to this point is to claim that what it is to possess a concept, like the concept of a zebra, or the concept of looking like a zebra, is to have certain connections between one’s lexical module and one’s perceptual modules established such that the lexical module primes the perceptual module in a certain way. (Such a view might be most plausible for observable concepts.) On this view, the lexical-perceptual modular connections mentioned in the previous paragraphs, which I suggested there were non-cognitive, would be conceptual and hence, one might think, count as cognitive. If one could make a good case for this position, then the influence of a lexical system on the perceptual system would count as cognitive penetration. This view of concepts, which has affinity with empiricist views of concepts, is the subject of much debate. As a result, if one were to take this line, one would need to argue that this view of concepts was plausible—a tricky task. What this shows is that establishing the cognition/non-cognition boundary or the cognition/perception boundary is a very difficult thing to do. But one has to either (a) establish those boundaries if one wants to establish the existence of cognitive penetration or (b) show that something that clearly falls on the side of cognition affects something that very clearly falls on the side of perception. To my mind, Lupyan has not done (a) and the experiments about the influence of words on perception are not the sort of cases demanded by (b).

## The Argument Against Cognitive Penetration from Illusion

Lupyan claims that some people have argued that the existence of known illusions—where one’s non-veridical perceptual experience is not affected by one’s knowledge or belief that that is not how the world is—shows that perception is not cognitively penetrated. One might think that that is the case because one thinks that this is an example where one’s cognitive state does not influence one’s perception. I believe that Lupyan is right to reject this line of reasoning, but there is a quick route to seeing why it should be rejected which Lupyan doesn’t consider. Notice that for perception to be cognitively penetrable, not every perceptual experience or instance of early perceptual processing needs to be penetrated. Thus, the existence of some cases of a lack of penetration does not show that perception is never penetrated. Only one case in which perception is cognitively penetrated is required in order to show that perception can be cognitively penetrated. (See Macpherson 2012.)

What reason does Lupyan give for thinking that the persistence of illusions, in the face of knowledge or belief that the world is not the way one’s perceptual experience presents it to be, fails to show that perception is not cognitively penetrated? Lupyan argues that there is cognitive penetration in cases of known illusion but it is not clear to me that he establishes that. Consider Lupyan’s explanation of the Müller-Lyer illusion. He cites evidence from Howe and Purves (2005) that, in images, lines that have adornments closer to their centre are actually on average shorter than lines with adornments farther from their centre. He claims that the visual system uses this bit of information in producing a representation of the world and doesn’t let other bits of information, such as one’s belief that the lines are equal, which one might have formed because one has been told that they are or because one has measured them to be, over-rule the first bit of information.

Suppose that is true. How does that help to show there is cognitive penetration when looking at the Müller-Lyer illusion? I cannot see that it does. Lupyan’s explanation suggests that one’s beliefs about the way the world is—one’s beliefs about the actual lengths of the lines—do not on this occasion influence perception. Might Lupyan be thinking that the information that lines that have adornments closer to their centre are on average shorter than lines with adornments farther from their centre is information that is in one’s cognitive system, and that is affecting one’s perception? If that were right, then cognitive penetration would be occurring. But what guarantee is there that that piece of information is in the cognitive system, rather than a piece of information stored in the low-level visual system? In fact, given that this information is not typically one of our beliefs about the world—as evidenced by the fact that research was needed to be done to discover that this was true—there is reason to think that this information is not typically in the cognitive system. Thus, it is unclear to me why Lupyan thinks that cognitive penetration is occurring in this case.

There are a variety of other claims that Lupyan makes concerning this case that I also do not understand—or understand their significance in his explanation of why cognitive penetration is taking place in the case of persisting known illusions:

(I) Does Lupyan think the Müller -Lyer is not an illusion or that the very concept of illusion should be altered or abandoned? Why exactly, and how does this help to show that cognitive penetration is occurring?
(II) What exactly is it to “minimise global prediction error” or to “represent a globally optimal solution”? Why would perception doing this show that there was cognitive penetration?
(III) Why should we think that in the case of the Müller-Lyer illusion the “bottom-up input is too uncertain [with respect to the length of the lines] (you need to put a ruler to it!)”, rather than think that the bottom-up input is perfectly certain and it is the mixed product of the top-down and bottom-up processing that is uncertain?
(IV) Why couldn’t the visual system be flexible and alter the weight that it gives to the hypothesis that lines with adornments closer to the centre are likely to be shorter than those with adornments further from the centre? It could do so when faced with very strong evidence from measuring that they are they are not, while holding that hypothesis to be typically true true and to apply to other cases. Why would this be “maladaptive” or “breaking the rest of vision”? It would seem to be a rather optimal solution.

Answers to these questions would help to clarify Lupyan’s thoughts about persisting illusions.

## Attention

In this section, I would like to simply echo the remarks that Lupyan makes about attention with respect to cognitive penetration.

Those who think that perception is not cognitively penetrated allow that there can be modulation of the output of early vision or perceptual experience by attention, where one’s attention is driven by cognition. That would be reasonable if attention simply selected which region of space should be visually processed: akin to moving one’s eyes or head. Perhaps some forms of attention are like that. However, as Lupyan rightly points out, some forms of attention that seem to affect experience are not like that. For example, there can be attention to some features rather than others within an area, not just selection of an area. See Macpherson (2012) for an example concerning colour. If that is right, then some attentional effects are candidates for being instances of cognitive penetration, and so those who deny the existence of cognitive penetration need to think more carefully about how to deal with such cases.

## The Relationship Between Predictive Coding and Cognitive Penetration

Recall that the main aim of Lupyan’s paper is to spell out the relationship between the thesis that the predictive coding model of perception is true and the thesis that cognitive penetration occurs. I believe that the nature of this relationship depends, in a highly sensitive manner, on the exact version of the predictive coding model of perception that one considers. Let me explain.

The most minimal statement of the predictive coding model that I can envisage would consist of three claims:

(A) the brain produces top-down generative models or representations of the world that are predictions of how the world is, drawing on many high-level processing levels;
(B) those representations are then modified bottom-up by incoming, lower-level processing, top-down by further high-level processing, and perhaps sideways by other sensory modalities;
(C) the resulting, “winning” representation is to be identified with perceptual experience.

Stated in this minimal fashion, predictive coding is consistent with cognitive penetration—both the penetration of early vision and the penetration of perceptual experience. But it is also consistent with cognitive penetration (of early vision and of perceptual experience) never occurring. And it is also consistent with cognitive penetration (of early vision and of perceptual experience) sometimes occurring. Of course, one could make a more specific statement of what one takes the predictive coding model to be. For example, one could specify that the high-level processing levels in (A) and (B) will:

(α): never involve cognitive states (such as beliefs, desires, and goals);
(β): sometimes involve cognitive states;
(χ): always involve cognitive states.

These precisifications will place constraints on the relationship between predictive coding and cognitive penetration. (α) entails that predictive coding is not consistent with cognitive penetration—neither the penetration of early vision nor the penetration of perceptual experience (so long as predictive coding is the only thing going on in the brain). (β) entails that predictive coding is consistent with cognitive penetration of experience, but that cognitive penetration of experience need not always occur. (χ) entails that cognitive penetration of experience always occurs. Whether (β) is consistent with cognitive penetration of early vision, or whether (χ) is consistent with or entails that cognitive penetration of early vision always occurs, is not yet settled by the considerations already adduced. Those questions will depend on a further fact: whether the top-down formation of representations affects the early visual system. It could be that the early visual system only provides a bottom-up error signal and is never affected by top-down processing. Or it could be that sometimes or always the top-down formation of representations affects the early visual system. This question can only be settled by both empirical enquiry, and detailed consideration on what one should take the early visual system to be, if one adheres to the predictive coding model. Thus, interestingly, settling the question of the relationship between the thesis that predictive coding is the correct account of perception and the thesis that cognitive penetration of experience occurs, does not by itself, in every instance, settle the question of the relationship between the thesis that predictive coding is the correct account of perception and the thesis that cognitive penetration of early vision occurs.

Which of these precisifications those who advocate predictive coding actually endorse is not entirely clear to me. I believe that some, including Lupyan, advocate (β) or (χ). (And while these entail, respectively, that cognitive penetration of experience sometimes occurs and that cognitive penetration of experience always occurs, further details have to be provided in order to determine whether these views would endorse the cognitive penetration of early vision happening sometimes or always.) But I believe that some could think (α)—perhaps on the grounds that on their model of the mind there is no distinction to be drawn between perception and cognition, and so one has to radically rethink the categories that folk psychology and current scientific psychology classifies mental and/or brain states. Certainly, each of the types of predictive coding model that I have discussed above are possible theories that one might endorse. Predictive coding theorists should consider explicitly specifying which of these theories they hold.

## Conclusion

Lupyan’s paper considers a great deal of evidence in favour of the idea that cognitive penetration occurs. I too have argued that it does; however, in this paper, I have questioned whether the evidence that Lupyan presents always allows him to reach this conclusion.

I argued that Lupyan needs to consider whether he wishes to endorse Pylyshyn’s definition of cognitive penetration because, on the one hand, the definition does not seem sufficient. On the other hand, given that Lupyan wishes to endorse the predictive coding model of perception, evidence about whether perceptual experience is affected by cognition does not entail that early vision is affected by cognition. I think that Lupyan would be better off arguing for the cognitive penetration of perceptual experience itself.

Second, I questioned whether Lupyan establishes that all cases of cross-modal effects are cases of cognitive penetration. In spelling out the fact that cross-modal effects and cognitive penetration are compatible, I articulated a form of cognitive penetration that has heretofore not been posited. Moreover, I claimed that, on the predictive coding model of perception, it seemed possible that cognition might not, on occasion, produce a prediction of how the world would be. Arguably, in such circumstances, cross-modal effects might exist without cognitive penetration occurring.

Third, I questioned whether Lupyan puts forward convincing evidence concerning whether categorical knowledge and language affect perception in a way that conclusively shows that cognitive penetration occurs. I argued that it was not obvious that he showed that the effects were really driven by cognition, rather than a lower-level lexical system.

Fourth, I queried Lupyan’s reply to the argument against cognitive penetration from illusion. I suggested that he misses a straightforward reply that shows the argument to be unsound. Moreover, I also examined his reply, and claimed that it is not adequate.

Fifth, I agreed with Lupyan’s comments about attention. Those who deny that there is cognitive penetration have work to do in examining forms of attention that they have heretofore ignored.

Finally, I spelled out the relationship between predictive coding and cognitive penetration arguing that different precisifications of predictive coding would lead to different answers as to what that relationship was ranging from the idea that predictive coding entails that cognitive penetration (either of experience or of early vision) never occurs, that it sometimes occurs, and that it always occurs.

## Footnotes

1 Note that, for comparison, in Macpherson (2012) I proposed another form of indirect cognitive penetration—indirect because it takes place via an intermediate state. In that instance it was via a state of imagination.

2 This position and Fodor’s argument for it was first brought to my attention by Bitter (unpublished).
